# Notes and News

**Published:** 1904-10-29

**Authors:** 


### NOTES AND NEWS.

Lady Rothschild visited the Epileptic Colony at Chalfont St. Peter last week to open the new administrative buildings, the gift of Mr. Passmore Edwards, and cost £18,500.

A scheme has been initiated to establish a Home of Recovery, situated in the country, to act as an auxiliary to surgical treatment in the London hospitals. The proposal is that patients whose healing is protracted should have the opportunity of trying the benefit of fresh air.

The new medical schools at the University of Dundee, opened by Lord Balfour of Burleigh at the beginning of the month, cost £20,000. The schools contain a very fine dissecting-room, equipped completely for the study of anatomy. There will be museums fitted for all branches of medical study, and the accommodation is for about 150 students.

The gold medal of the British Medical Association has been awarded to Sir Constantine Holman for his distinguished services to the British Medical Association, through the South-Eastern Branch, on the Central Council as treasurer and as vice-president; and, further, for his lifelong and successful labours for the improvement of the financial conditions of the medical charities.

A cottage hospital has been opened at Tredegar by Lord Tredegar, situated in the park which he had previously presented to the town. The accommodation for 11 patients includes an operating-room, with anæsthetic room, dispensary, committee-room, and staff and domestic quarters. The completeness of the arrangements for so small a number of patients accounts for the relatively high expenditure of £300 per bed. An interesting feature in connection with the establishment of this small institution is that the scheme was drawn up with the co-operation of a working men's committee, and that 80 per cent. of the working-men population have promised a half-penny per week towards its maintenance.

Professor Robert Koch is to be the recipient of the Nobel Prize in medicine this year.

The new Maternity Hospital for Belfast will be opened by Countess Grosvenor on November 7th.

The Fitzpatrick Lectures before the Royal College of Physicians will be given on November 8th and 10th by Dr. J. Payne, who will lecture on Anglo-Norman Medicine. The Bradshaw Lecture will be given by Dr. F. Foord Caiger on November 15th on Enteric Fever.

The appeal in aid of the Queen Alexandra Sanatorium at Davos made by the Princess Christian is a happy one. It is directed to the principal cities and towns of the United Kingdom, with the object of securing subscribers from each locality. It is fitting that all should co-operate, as patients from every community will be eligible for treatment.

The Rev. Prebendary Ingram asks us to draw the attention of our readers to a useful institution known as St. John's House of Rest, at Mentone, which is open for the reception of clergy and laymen needing rest and change at a nominal charge. The committee occasionally help towards travelling expenses when the case is very necessitous.

The Eversfield Hospital for Consumption and Diseases of the Chest, St. Leonards, is now reopened. It had been closed for cleaning and alterations. This is an institution, supported mainly by voluntary subscriptions, which receives patients for small payments. The cost of alterations amounted to £650, of which only about £400 has yet been obtained.

At the last meeting of the Sheffield Royal Hospital Mr. Harry Fisher complained of the abuse of recommendations by people who had no right to gratuitous hospital relief, and mentioned the case of a lady who called in her carriage to ask for a recommendation for her sister. He advocated proper inquiry, and pointed out the injury done to the medical profession by the admission of unsuitable patients. The Lord Mayor suggested that the attention of subscribers should be drawn to this matter in the annual report.

Dr. Léon Dufour, of Fécamp (Seine-Inferieure), France, acting on behalf of French directors of "Gouttes de Lait," has invited all medical men engaged or interested in the work of these organisations to attend a three days' congress in Fécamp, from October 28th to 30th inclusive. It is intended to form the individual charities into some sort of union, in touch with each other through a central organisation. The promoters of the congress anticipate more rapid progress both in method and in the increase of establishments by such a union. The programme is to be as follows:—October 28th: opening ceremony; congress. October 29th: congress; evening banquet. October 30th: morning visit to Havre "Goutte de Lait"; afternoon visit to Rouen "Goutte de Lait." Among the subjects to be discussed are "Gastro-Enteritis and Sterilised Milk" and "Laws Regulating the Sale of Milk."

